# A functional spleen contributes to afucosylated IgG in humans

**DOI:** 10.1038/s41598-021-03196-w

**Published:** 2021-12-15

**Authors:** Iwona Wojcik, David E. Schmidt, Lisa A. de Neef, Minke A. E. Rab, Bob Meek, Okke de Weerdt, Manfred Wuhrer, C. Ellen van der Schoot, Jaap J. Zwaginga, Masja de Haas, David Falck, Gestur Vidarsson

**Affiliations:** 1 Center for Proteomics and Metabolomics, Leiden University Medical Center, Leiden, The Netherlands; 2 Glycoscience Research Laboratory, Genos Ltd., Zagreb, Croatia; 3 Department of Experimental Immunohematology, Sanquin, Amsterdam, The Netherlands; 4 Department of Central Diagnostic Laboratory-Research, University Medical Center Utrecht, Utrecht University, Utrecht, The Netherlands; 5 Department of Hematology, University Medical Center Utrecht, Utrecht University, Utrecht, The Netherlands; 6 Department of Medical Microbiology and Immunology, St. Antonius Hospital, Nieuwegein, The Netherlands; 7 Department of Internal Medicine, St. Antonius Hospital, Nieuwegein, The Netherlands; 8 Center for Clinical Transfusion Research, Sanquin Research, Leiden, The Netherlands; 9 Department of Immune Hematology and Blood Transfusion, Leiden University Medical Center, Leiden, The Netherlands; 10 Department of Immunohematology Diagnostics, Sanquin, Amsterdam, The Netherlands; 11 Landsteiner Laboratory, Amsterdam UMC, University of Amsterdam, Amsterdam, The Netherlands

**Keywords:** Glycobiology, Immunological disorders

## Abstract

As a lymphoid organ, the spleen hosts a wide range of immune cell populations, which not only remove blood-borne antigens, but also generate and regulate antigen-specific immune responses. In particular, the splenic microenvironment has been demonstrated to play a prominent role in adaptive immune responses to enveloped viral infections and alloantigens. During both types of immunizations, antigen-specific immunoglobulins G (IgGs) have been characterized by the reduced amount of fucose present on N-linked glycans of the fragment crystallizable (Fc) region. These glycans are essential for mediating the induction of immune effector functions. Therefore, we hypothesized that a spleen may modulate humoral responses and serve as a preferential site for afucosylated IgG responses, which potentially play a role in immune thrombocytopenia (ITP) pathogenesis. To determine the role of the spleen in IgG-Fc glycosylation, we performed IgG subclass-specific liquid chromatography–mass spectrometry (LC–MS) analysis of Fc glycosylation in a large cohort of individuals splenectomized due to trauma, due to ITP, or spherocytosis. IgG-Fc fucosylation was consistently increased after splenectomy, while no effects for IgG-Fc galactosylation and sialylation were observed. An increase in IgG1- and IgG2/3-Fc fucosylation level upon splenectomy has been reported here for the first time, suggesting that immune responses occurring in the spleen may be particularly prone to generate afucosylated IgG responses. Surprisingly, the level of total IgG-Fc fucosylation was decreased in ITP patients compared to healthy controls. Overall, our results suggest a yet unrecognized role of the spleen in either the induction or maintenance of afucosylated IgG responses by B cells.

## Introduction

The spleen is a key immune organ of the human body, enabling close interaction of the innate and adaptive immune system^1^. The spleen acts as a phagocytic blood filter, as splenic macrophages remove blood-borne aged cells and microorganisms. Moreover, the spleen is also a site of antibody production by long-lived memory B cells, plasmablasts and plasma cells^1,2^. In the lymphoid white pulp, T- and B-cells interact with antigen-presenting cells such as dendritic cells or marginal zone B-cells^1,3^. In line with these functions, surgical splenectomy is associated with several negative consequences. The reduced function or absence of the spleen can lead to infectious complications, impaired erythrocyte and iron recycling, and thromboembolic disease^4^. Immunologically, immunoglobulin M (IgM) memory B cells and switched memory B cells are markedly reduced after splenectomy^4–6^. Serum IgM is reduced following splenectomy, whereas immunoglobulin G (IgG) and immunoglobulin A (IgA) are similar to control values^7^. Generally, splenectomized individuals seem to be particularly sensitive to a drop in levels of certain antigen-specific antibodies, especially to T-cell independent antigens, such as pneumococcal polysaccharides. This is probably as the spleen is not only important for the formation of antibody-responses to both T-cell independent antigens and antigens in the blood, but also for the maintenance of those responses^3^. In line with this, the number of anti-pneumococcal polysaccharide IgM and IgG memory B cells are reduced following splenectomy^8^. For this reason, pneumococcal vaccination, especially with protein conjugated vaccine, is highly recommended before splenectomy^9^.

Antibodies represent a major effector mechanism of the adaptive immune system. Besides Fab-based affinity maturation, the effector functions of IgG antibodies are modified by N-linked glycosylation of the antibody Fc (fragment crystallizable) portion at position 297. The exact composition of this glycan branch can be affected in an antigen-specific manner^10–13^. This, in turn, modifies the binding to complement component C1q and to the IgG Fc gamma receptor III (FcγRIII) on immune cells, and determines the complement activity and antibody-dependent cellular cytotoxicity^14^. Afucosylated IgG display enhanced binding to FcγRIII expressed on CD16^+^ monocytes, macrophages, neutrophils and natural killer (NK) cells causing increased antibody-dependent cellular cytotoxicity^14^. An increase in the Fc galactosylation of IgG leads to increased C1q-mediated complement activation^14,15^.

In autoimmune diseases, total serum IgG-Fc glycosylation is skewed, mostly with lowered galactosylation, and this is associated with increased disease progression, activity and symptoms severity^16–19^. Why this occurs is unclear, as elevated galactosylation of antigen-specific IgG is known to elevate their complement activity potential^14,15,20^. However, a possible explanation of the association between the lowered total IgG galactosylation and autoimmune diseases, is that the decrease in total IgG galactosylation seems to increase systemic inflammation^19,20^. Observations in Guillain-Barré syndrome and Kawasaki disease suggest that pre-treatment levels and normalization of Fc galactosylation and sialylation after treatment are associated with recovery^21,22^. Interestingly, we found no biologically significant changes in IgG-Fc glycosylation were observed after CD20-targeted rituximab treatment in immune thrombocytopenia (ITP)^23^.

For alloimmune responses, that is foreign antigens on cells such as platelets and anti-red blood cell antigens (RBC) in either pregnancy or after transfusion, we previously described a general reduction of antigen-specific IgG-Fc fucosylation, such as for anti-D and anti-Human Platelet Antigen-1a (HPA-1a) antibodies^24–26^. Importantly, immunization to these alloantigens, also generally require a functional spleen^27^, suggesting that immune responses occurring in the spleen may be particularly prone to generate afucosylated IgG responses. Similar afucosylated antigen-specific IgG responses have been observed in virus-specific IgG responses, such as for dengue virus, HIV, cytomegalovirus (CMV), hepatitis B virus (HBV), measles virus, mumps virus and severe acute respiratory syndrome coronavirus 2 (SARS-CoV-2)^11–13,28,29^, and now recently shown by us to be a hallmark of enveloped viral infections, a response that is mimicked by alloimmunizations^11^. In theory, IgG afucosylation results in a strong humoral immune response, targeting FcγRIIIa and FcγRIIIb, potentially giving strong myeloid FcγR-mediated responses^30^ that can ultimately be particularly protective as proposed for HIV^31^. On the other hand, this strong reaction can also lead to exaggerated myeloid response with elevated proinflammatory cytokine production culminating in adverse clinical reactions, as seen in Dengue and COVID-19 responses^11,28,32^.

The reduction in fucosylation seen in immune response to enveloped viral infections and alloimmune mediated diseases is particularly strong to CMV and many of the anti-red blood cell antigens (e.g. Rhesus D) and human platelet antigens. What these antigen responses have in common is a dominant systemic response likely to occur in the spleen^27^.

In the present study, we investigated the role of the spleen on IgG-Fc glycosylation in a large cohort of individuals splenectomized due to trauma, or due to immune thrombocytopenia, or spherocytosis, to test the hypothesis that the spleen is a primary driver of afucosylated IgG responses in humans.

## Results

We studied the impact of splenectomy on IgG-Fc N-linked glycosylation by comparing patients who had undergone splenectomy to control groups with an intact spleen. A proportion of individuals were splenectomized after trauma (n = 38), but were otherwise healthy, whereas others were splenectomized due to hematological disease (ITP, n = 35; spherocytosis, n = 16) (Table 1). All of the subgroups of healthy controls and ITP patients were comparable for age and sex distribution thus reducing the age- and sex-depended differences in IgG glycosylation. This allowed us to test our hypothesis of the effect of the spleen on IgG glycosylation in both relatively healthy individuals and individuals with an autoimmune disease.

**Table 1:** Demographics of patients splenectomized either due to trauma or hematological diseases and non-splenectomized matched controls.

	Healthy		ITP		Spherocytosis	
	Trauma splenectomized	Healthy controls	ITP splenectomized	ITP controls	Spherocytosis splenectomized	Healthy controls
N	38	76	35	57	16	32
**Sex, n (%)**						
Female	18 (47)	36 (47)	14 (40)	24 (42)	7 (44)	14 (44)
Male	20 (53)	40 (53)	21 (60)	33 (58)	9 (56)	18 (56)
Median age, years [IQR]	50 [39–56]	50 [39–56]	52 [44–62]	54 [45–62]	38 [30–53]	38 [30–53]
Median interval*, years [range]	29 [6–55]	–	HOVON64: 12 [0.2–29] Utrecht: 19 [5–51]	–	28 [5–50]	–

*Median interval between splenectomy and blood sampling.

### Measurement of IgG-Fc glycosylation

**Figure 1:**
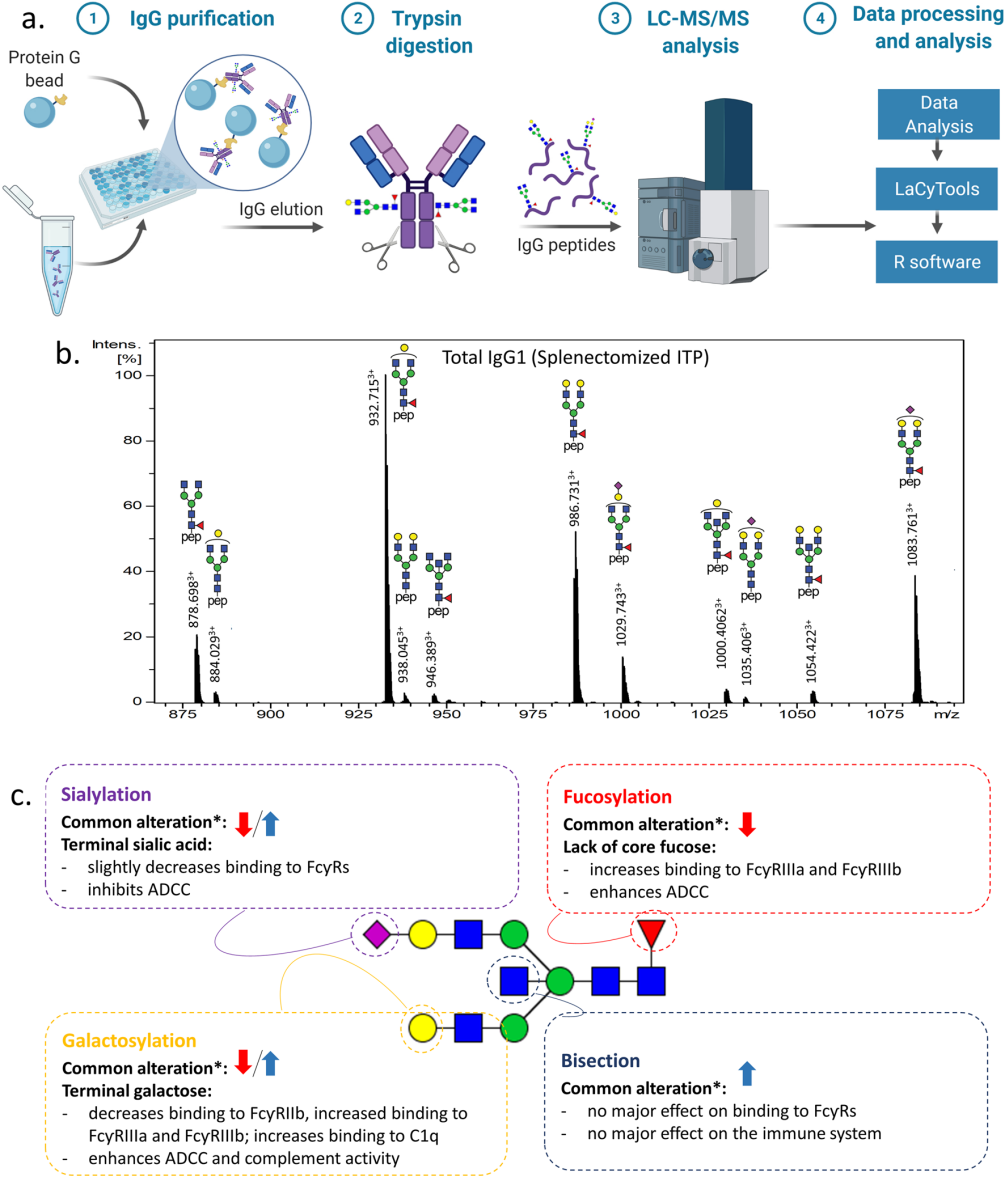
LC–MS analysis of IgG-Fc glycopeptides. (**A**) Schematic workflow. The figure was created with BioRender.com (**B**) Representative LC–MS summed mass spectrum of IgG1 tryptic glycopeptides obtained from a 43 year-old splenectomized ITP male. Annotated are the 11 most abundant IgG1 glycopeptides. Green circle: mannose, yellow circle: galactose, blue square: N-acetylglucosamine, red triangle: fucose, pink diamond: N-acetylneuraminic acid. (**C**) Scheme of the IgG-Fc glycan structure, common disease-related changes in total IgG and their immunological implications. It shows derived glycosylation traits representing common glycosylation features shared by glycan composition: sialylation—sialylation per antenna of biantennary glycans, fucosylation—fraction of fucosylated glycans, galactosylation—galactosylation per antenna of biantennary glycans, bisection—fraction of glycans with bisecting N-acetylhexosamine. For detailed calculation per IgG subclass see Supplementary Table S2. *Down arrow refers to a commonly observed lower and up arrow to higher proportion of the corresponding IgG glycosylation trait in patients suffering from autoimmunological, infectious and inflammatory diseases compared to healthy controls; ADCC—antibody-depended cellular cytotoxicity*.*

Total IgG was affinity purified from 254 patient plasma samples, subjected to tryptic digestion, and the resulting IgG Asn297-Fc glycopeptides were analyzed by liquid chromatography–mass spectrometry (LC–MS) (Fig. 1A). Tryptic digestion of all four IgG subclasses (IgG1, IgG2, IgG3, IgG4) resulted in distinct peptide moieties (IgG1: EEQYNSTYR, IgG2/3: EEQFNSTFR, IgG4: EEQFNSTYR). Thus, IgG-Fc glycopeptides were separated and glycosylation patterns of individual subclasses IgG1, IgG4 as well as collective Fc glycosylation profiles of IgG2/IgG3 were analyzed by LC–MS. An example of an LC–MS sum spectrum of total IgG1 glycopeptides can be seen in Fig. 1B**.** Inability to distinguish between IgG2 and IgG3 glycosylation by our profiling method is due to the fact that the amino acid sequence of the tryptic peptide including the N297-glycosylation site is identical for both IgG subclasses (EEQFNSTFR)^33,34^. For all samples, 37 glycoforms positively passed the analyte curation, 19, 10 and 8 glycoforms for IgG1, IgG2/3 and IgG4, respectively (see Supplementary Table S1). For further analysis, derived glycosylation traits were calculated, including bisection, fucosylation, galactosylation and sialylation (Fig. 1C, see Supplementary Table S2 for the calculations of the derived traits). It should be noted that we could not assess the minor amount of IgG4 afucosylation which is a common limitation of the applied method.

### The impact of splenectomy on IgG-Fc glycosylation

**Figure 2:**
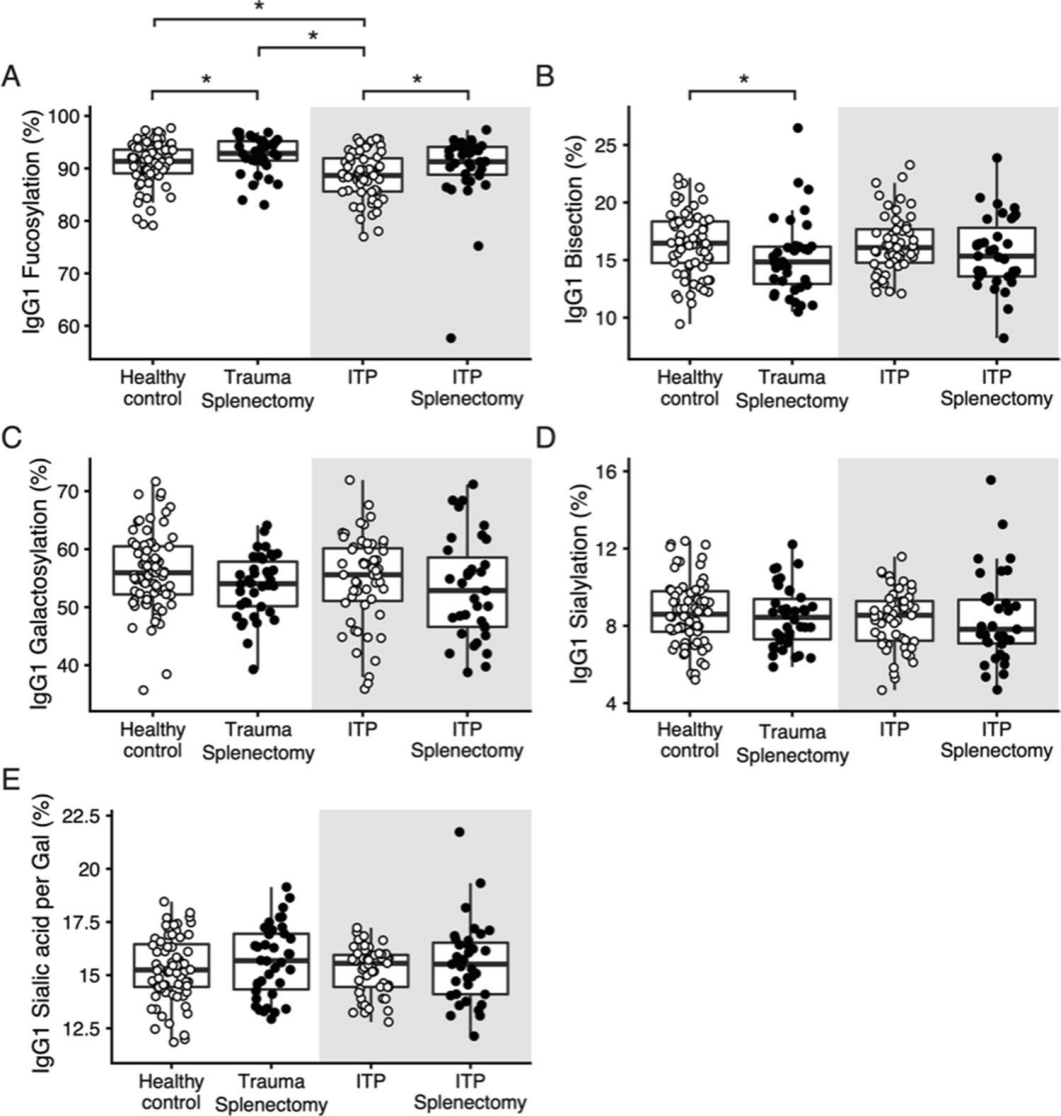
Effect of splenectomy on IgG1-Fc glycosylation in healthy individuals and ITP patients. Data are shown for healthy individuals with (Healthy Control, *n* = 38) or without splenectomy (Trauma Splenectomy, *n* = 76) and ITP patients with (ITP Splenectomy, *n* = 35) or without splenectomy (ITP, *n* = 57), matched for age and sex. Compared to the respective controls, IgG1-Fc fucosylation was higher in splenectomized and otherwise healthy individuals (*P* = 0.026; Wilcoxon-rank sum test), as well as in splenectomized ITP patients (*P* = 0.017; Wilcoxon-rank sum test). Regarding IgG1-Fc bisection, otherwise healthy individuals showed lower levels (*P* = 0.008; Wilcoxon-rank sum test), whereas in splenectomized ITP patients this difference was less prominent and did not result in a finding. ITP patients have reduced IgG1-Fc fucosylation compared to healthy controls (*P* = 0.017; Kruskal–Wallis rank sum test followed by a post-hoc Nemenyi test for pairwise comparison). All other glycosylation features were not significantly different between the groups. *P-values are given for a Wilcoxon-rank sum test, *P*-values < 0.05; ns, not significant.

**Figure 3:**
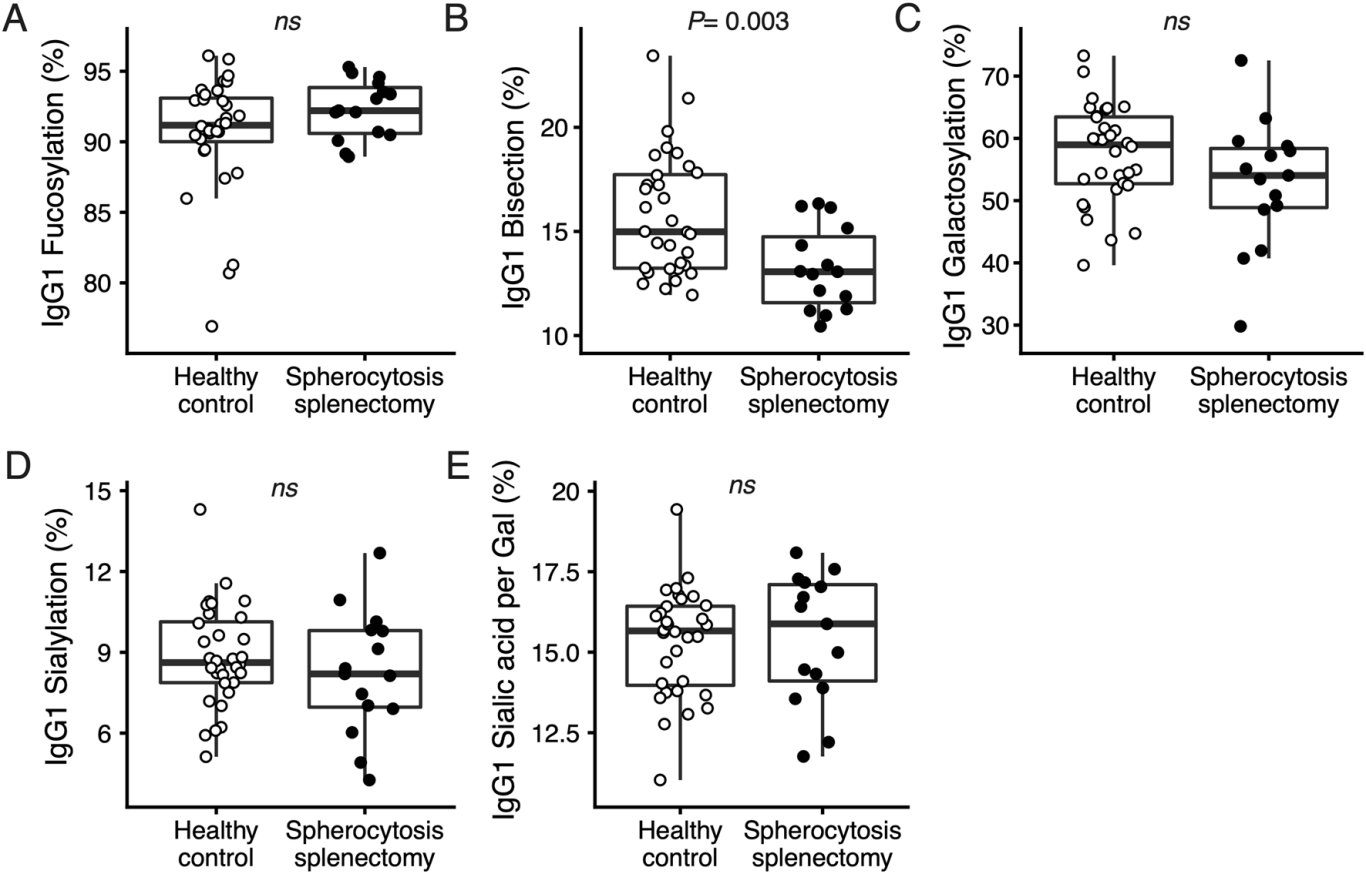
Effect of spherocytosis-associated splenectomy on IgG1-Fc glycosylation. Data are shown for healthy individuals without splenectomy (Healthy control, *n* = 32) and spherocytosis patients with splenectomy (Spherocytosis splenectomy, *n* = 16). Compared to the respective controls, spherocytosis-associated splenectomy individuals showed lower levels of IgG1-Fc bisection (*P* = 0.003; Wilcoxon-rank sum test), and trend towards increased IgG1-Fc fucosylation. All other glycosylation features were not significantly different between the groups.

To evaluate the impact of splenectomy on total IgG-Fc glycosylation, we compared samples from three different patient groups with splenectomy with age- and sex-matched respective controls with an intact spleen. The three different splenectomized patient groups were 1) trauma-associated 2) ITP patients and 3) spherocytosis patients. For trauma splenectomized individuals, we observed a significantly higher median IgG1 fucosylation and IgG2/3 fucosylation when compared to healthy controls (Table 2; Fig. 2A for IgG1; Supplementary Fig. S2A for IgG2/3). Likewise, the analysis of IgG-Fc glycosylation in splenectomized ITP patients revealed a higher median fucosylation for both IgG1 and IgG2/3, compared to ITP controls. For spherocytosis patients, we did not observe any significant differences in the level of IgG-Fc fucosylation in splenectomized individuals compared to healthy individuals (Fig. 3A). Bisection was significantly decreased for the trauma group and spherocytosis compared to healthy controls for both IgG1 and IgG2/3 (Table 2, Figs. 2B and 3B, see Supplementary Fig. S2B for IgG2/3 data). No differences were observed for any other glycan traits in any IgG subclass (Figs. 2C–E and 3C–E, Supplementary Figs. S2C–E and S3C–E).

**Table 2:** Glycosylation comparison of two different patient groups (healthy and ITP) with splenectomy and age- and sex-matched respective controls with an intact spleen.

IgG subclass	Derived trait	Healthy				
		Splenectomized		Controls		*P*-value
		Median	IRQ	Median	IRQ	
IgG1	fucosylation	92.9	91.5–95.2	91.4	89.1–93.6	0.026
	bisection	14.8	12.9–16.2	16.5	14.7–18.4	0.008
IgG2/3	fucosylation	99.8	99.7–99.8	99.7	99.5–99.8	0.003
	bisection	11.9	10.4–13.4	12.8	11.7–15.8	0.042
		**ITP**				
IgG1	fucosylation	91.3	88.8–94.1	88.7	85.6–91.9	0.017
IgG2/3	fucosylation	99.6	99.5–99.8	99.5	99.4–99.7	0.016

*P*-values are given for a Wilcoxon-rank sum test. Only significant differences in IgG-Fc glycosylation (*P* < 0.05) are shown.

### ITP patients and healthy controls differ in IgG fucosylation

Comparison of the IgG-Fc glycosylation of ITP patients with healthy controls showed that the ITP patients had significantly lower median IgG1 fucosylation compared to healthy controls (Fig. 2A). The splenectomy resulted in an elevated level of IgG1 fucosylation in ITP patients, with undistinguishable IgG1 fucosylation between splenectomized ITP patients and healthy individuals. Moreover, ITP patients had a lower IgG1-Fc fucosylation than trauma-related splenectomized patients. No other significant changes were observed comparing healthy controls for any IgG subclass or glycosylation trait (Fig. 2B–E, Supplementary Fig. S2B–E for IgG2/3, Supplementary Fig. S3A–E for IgG4).

## Discussion

Splenectomy performed in ITP patients and healthy individuals due to trauma represents a unique opportunity to study the role of the spleen in control of IgG glycosylation. The key finding of our study is an increase in IgG1 fucosylation and in IgG2/3 fucosylation upon splenectomy. Although these changes in total plasma IgG are minor, with total IgG only containing ~ 6% of afucosylated IgG, they are likely to be larger and give rise to significant biological effects when looking at the antigen-specific level. For example, the ability to enhance the inflammatory response by afucosylated antigen-specific IgG was observed in fetal or neonatal alloimmune thrombocytopenia (FNAIT), dengue virus infection, COVID-19^11,26,29,32^. Among all four patient groups, the otherwise healthy individuals after trauma-associated splenectomy showed the highest level of IgG1-Fc fucosylation. An increase in IgG-Fc fucosylation due to splenectomy was found in both healthy individuals as well as in ITP patients. Similar changes were seen in spherocytosis. This could either be due to, support of short lived afucosylated IgG responses generated by B cells in the spleen, or the spleen is a reservoir for long lived plasma cells^2^. Possibly, the less pronounced effect on IgG2/3 is because IgG2 is the more abundant subclass of these two (~ 32% vs 4%, respectively)^34^, and because IgG2 fucosylation is generally very high, which is in line with the predominant IgG2 response to encapsulated bacteria which do not induce afucosylated IgG responses^11,35,36^. However, IgG3 responses most often occur simultaneously with IgG1 responses, and the level of fucosylation of both IgG1 and IgG3 generally seem to go hand in hand^37^. It is therefore likely that the lowered effect of splenectomy on IgG2/3 afucosylation is solely due to the IgG3 fraction. Together, this points towards a pronounced role of IgG1 and IgG3 in platelet clearance compared to other IgG subclasses. Due to the prominent role and an inability to distinguish the effect size of fucosylation changes between IgG3 and IgG2, our discussion applies mainly to IgG1.

Without any intervention or emerging pathology, total IgG-Fc fucosylation is largely stable throughout the adult life^38^. The precise regulation of the IgG-Fc glycosylation, and fucosylation in particular, remains still largely unelucidated. Shortly after birth, neonates make exclusively fucosylated antibodies, but appear to gradually acquire afucosylated IgG until adulthood reaching the level of 96% of IgG-Fc fucosylation^38^. Recently, we found that only antigens expressed on the surface of host cells, such as alloantigens and proteins of enveloped viruses found on cell surfaces, seem to induce afucosylated IgG responses^11^. Based on this, it is tempting to hypothesize that the gradual accumulation of afucosylated IgG seen during childhood, is the accumulation of antigen-specific IgG to enveloped viruses. However, the tendency to generate afucosylated IgG varies vastly between targets and between individuals, with IgG1 responses to cytomegalovirus being generally low and stable in time, while responses to SARS-CoV-2 start with relatively high level of IgG1 fucosylation in most individuals. However, in some patients afucosylated IgG1 is observed at seroconversion, which is associated with enhanced pathologies due to excessive inflammation in COVID-19, those responses quickly revert to fucosylated IgG in a matter of weeks^11,32^. Conversely, alloantibodies specific for HPA-1a, causing FNAIT, or to RBC show remarkable low levels of fucosylation that are extremely stable for more than 10 years^10,24,26^. All in all, these disparate type of afucosylated IgG responses seem to favor both possible roles for the spleen in generating and maintaining IgG afucosylation. The afucosylation of anti-platelet antibodies has been recognized as a very important factor enhancing platelet-specific IgG effector functions through elevated affinity to FcγRIIIa/b and finally target destruction and production of proinflammatory cytokines^25,32,39^. The spleen, second to the liver, is recognized as one of the most important organs for removal of IgG-opsonized cells from the blood, with monocytes expressing FcγRIIIa being particularly abundant^40,41^. Therefore, splenectomy has typically been seen as a resort focusing on removing the organ responsible for and thus preventing degradation of cells in diseases like ITP. However, it is important to realize that splenectomy not only removes an important myeloid compartment responsible of removing IgG-opsonized platelets, but also removes the major site of auto-antibody production in ITP. The splenectomy removes many autoantibody-producing cells, especially in cases of autoreactive B cells to platelets in ITP^42,43^. Accordingly, this results in the profound elimination of anti-platelet IgG production in a splenectomized mouse model for ITP^44^. As opposed to anti-platelet IgG levels, total plasma IgG levels were not affected by splenectomy^45^. Interestingly, when targeting B cells systemically with Rituximab (anti-CD20) in ITP, afucosylated antibodies are not specifically reduced^23^, which is in line with the fact that long lived plasma cells do not express CD20^46^. A limitation in the present study is that we did not investigate antigen-specific antibodies individually. Monitoring platelet autoantibody IgG-Fc glycosylation levels should provide further information for elucidating the mechanism of afucosylated responses in the spleen. In addition, the effect of splenectomy on composition of antigen-specific plasma cells in the bone-marrow would be valuable in a future study.

In this study, total IgG1 and IgG2/3 purified from plasma of ITP patients showed decreased fucosylation when compared to healthy individuals, while galactosylation, sialylation and bisection remained similar. In previous studies, we found a similar result and effect size, but this was not statistically significant due to lower power^23^. This finding in ITP is in contrast to many autoimmunological, inflammatory and infectious conditions, where mainly galactosylation and sialylation of total IgG1 have been described to be altered^19,47–49^. However, afucosylation of total IgG has recently been found to be elevated in autoimmune thyroid diseases^50^, similar to our ITP findings. This may suggest that the mechanism underlying the changes in IgG glycosylation in autoimmune diseases may vary depending on the disease etiology, target tissue, or the level of antigen-specific IgG glycosylation.

## Conclusions

Splenectomy has an effect on total plasma IgG-Fc glycosylation. ITP patients showed a lower level of total IgG-Fc fucosylation compared to healthy controls, implying that anti-platelet autoantibodies have an afucosylation phenotype in ITP, as demonstrated for FNAIT. In addition to removing a major organ responsible for removal of IgG-autoantibody opsonized cells in ITP, we also find a general splenectomy-driven increase in IgG-Fc fucosylation in humans. Altogether, our data indicate that the spleen is a significant site to generate or maintain afucosylated IgG responses.

## Methods

### Study participants

Plasma samples from individuals with splenectomy due to trauma, spherocytosis or immune thrombocytopenia were identified by regional general practitioners and a nationwide database^45^. The study was approved by the ethical review committee of St Antonius Hospital Nieuwegein, The Netherlands. Written informed consent was obtained from the study subjects. Further samples from splenectomized ITP patients were obtained from the HOVON64 study^51^. The study was approved by the ethical review committee of Academic Medical Center, Amsterdam, The Netherlands; subjects provided written informed consent. Age- and sex-matched control samples were obtained from healthy blood donors (Sanquin, Amsterdam, The Netherlands) and non-splenectomized ITP patients from the HOVON64 study^51^. All splenectomy samples were matched to two control samples. Patients from the HOVON64 study were splenectomized with a median interval of 12 years before blood sampling (range: 2 months–29 years). Patients from the Utrecht study were splenectomized with a median interval of 29 years before blood sampling (range: 6–55 years) for trauma, 19 years (range: 5–51 years) for ITP and 28 years (range: 5–50 years) for spherocytosis. Samples were handled according to national responsible use protocols (Federation of Dutch Medical Scientific Societies Code of Conduct for responsible use; www.federa.org). All samples and data were coded for storage and analysis.

### IgG purification and enzymatic digestion

All 254 plasma samples from splenectomized (n = 89) and non-splenectomized individuals (n = 165) were randomized through four 96-well plates, including 24 pooled plasma samples and 23 plasma standards (VisuCon_F control plasma; Affinity Biologicals Inc., Ancaster, ON, Canada). Total IgG (IgG1, IgG2, IgG3 and IgG4) was affinity-purified by protein G affinity beads (GE Healthcare, Uppsala, Sweden) and subjected to trypsin digestion in 96-well filter plates (0.7 mL wells, PE frit, Orochem, Naperville, IL) as described before^52^, see Methods in Supplementary Material. Obtained IgG glycopeptides were stored at − 20 °C until measurement.

### Mass spectrometric analysis and data processing

The total IgG glycopeptides were analyzed using an Ultimate 3000 RSLC nano-liquid chromatography system (Dionex/Thermo Fisher Scientific, Sunnyvale, CA) coupled to a Maxis Impact HD quadrupole time-of-flight (QTOF)-MS instrument (micrOTOF-Q; Bruker Daltonics, Bremen, Germany) as described previously^52^. Data were processed using the highly automated LaCyTools software as previously described^52,53^. Processing parameters and the software used to compile them are listed in the supporting information. Glycopeptide compositions were selected based on literature^54^ and manual inspection with Data Analysis (version 5.0; Bruker). They were verified or rejected using quality criteria provided by LaCyTools. Per IgG subclass, samples were checked for analyte quality and total signal intensity and extreme cases removed (see Supporting Information). Total area normalization was applied. Based on the normalized, directly measured N-glycan monosaccharide composition, the following derived glycosylation traits were calculated (Supplementary Table S2): fucosylation (fraction of fucosylated N*-*glycopeptides), galactosylation (fraction of galactoses per antennae of biantennary glycans), bisection (fraction of *N*-glycopeptides carrying a bisecting *N*-acetylglucosamine (GlcNAc)), sialylation (fraction of sialic acids per antennae of biantennary glycans) and sialic acid per galactose (fraction of sialic acids per galactose). Fucosylation for IgG4 could not be accurately assessed, since the exact mass of the minor IgG4 afucosylated N-glycopeptides overlapped with the more abundant IgG1 fucosylated N-glycopeptides (Supplementary Table S1). Moreover, the IgG4 fucosylated N-glycopeptides have the same exact mass as the afucosylated ones from IgG2/3. A study is characterized by a good analytical precision of the LC–MS method, which was assessed based on 24 replicate analysis of pool plasma standard throughout the cohort (Supplementary Fig. S1).

### Statistical analyses

Analyses were performed in R version 3.6.4 (R Core Team). As the distribution of normalized glycan species and the derived traits deviates significantly from normality, further statistical analysis was performed using non-parametric tests^55^. A non-parametric Wilcoxon-rank test was used to compare relative intensities of the IgG-Fc glycosylation traits between pairs (non-splenectomized and splenectomized). A two-sided *P*-value below 0.05 was considered significant. Statistical tests were only performed for IgG glycosylation traits, other data were reported descriptively. Differences in relative intensities of IgG-Fc glycosylation traits across all patient groups were evaluated for statistical significance using the non-parametric Kruskal Wallis rank sum test followed by a post-hoc Nemenyi test for pairwise comparison.

## Supplementary Information

Supplementary Information 1.
