# Supplementary material for: A functional spleen contributes to afucosylated IgG in humans
Source: Sci Rep. 2021 Dec 15;11:24045. doi: 10.1038/s41598-021-03196-w (PMC8674363; doi:10.1038/s41598-021-03196-w)
Supplement: Supplementary file 1 — Supplementary Information 1. [file 41598_2021_3196_MOESM1_ESM.docx]

**Supplementary Information**

**A functional spleen contributes to afucosylated IgG in humans**

**Iwona Wojcik^1,2^*, David Schmidt^3^*, Lisa A. de Neef^1^, Minke A.E. Rab^4,5^, Bob Meek^6^, Okke de Weerdt^7^, Manfred Wuhrer^1^, C. Ellen van der Schoot^3^, Jaap J. Zwaginga^8,9^, Masja de Haas^8,9,10^, David Falck^1^* and Gestur Vidarsson^3,11^***

^1^ Center for Proteomics and Metabolomics, Leiden University Medical Center, Leiden, The Netherlands

^2^ Glycoscience Research Laboratory, Genos Ltd., Zagreb, Croatia

^3^ Department of Experimental Immunohematology, Sanquin, Amsterdam, The Netherlands

^4^ Department of Central Diagnostic Laboratory-Research, University Medical Center Utrecht, Utrecht University, The Netherlands

^5^ Department of Hematology, University Medical Center Utrecht, Utrecht University, The Netherlands

^6^ Department of Medical Microbiology and Immunology, St. Antonius Hospital, Nieuwegein, The Netherlands

^7^ Department of Internal Medicine, St. Antonius Hospital, Nieuwegein, The Netherlands

^8^ Center for Clinical Transfusion Research, Sanquin Research, Leiden, The Netherlands

^9^ Department of Hematology, Leiden University Medical Center, Leiden, The Netherlands

^10^ Department of Immunohematology Diagnostics, Sanquin, Amsterdam, The Netherlands

^11^ Landsteiner Laboratory, Amsterdam UMC, University of Amsterdam, Amsterdam, The Netherlands

*Both authors contributed equally

Correspondence and request for materials should be addressed to Gestur Vidarsson (email: [g.vidarsson@sanquin.nl](mailto:g.vidarsson@sanquin.nl))

Alternative Corresponding Author: Iwona Wojcik (i.t.wojcik@lumc.nl)

**TABLE OF CONTENTS**

[Additional Experimental Details 2](#_Toc82080696)

[Materials 2](#_Toc82080697)

[IgG purification and enzymatic digestion 2](#_Toc82080698)

[Mass spectrometric analysis 2](#_Toc82080699)

[Data processing 3](#_Toc82080700)

[Supplementary Tables 4](#_Toc82080701)

[S1. The list of curated glycosylation compositions 4](#_Toc82080702)

[S2. Derived traits calculation 5](#_Toc82080703)

[Supplementary Figures 7](#_Toc82080704)

[S1. Repeatability of the method 7](#_Toc82080706)

[S2. Comparison of IgG2/3-Fc glycosylation profiles 8](#_Toc82080707)

[S3. Comparison of IgG4-Fc glycosylation profiles 9](#_Toc82080709)

[References 10](#_Toc82080710)

# **Additional Experimental Details**

## Materials

Ultra-pure deionized water (MQ) was generated by the Purelab Ultra, maintained at 18.2 MΩ (Veolia Water Technologies Netherlands B.V., Ede, The Netherlands) and used throughout the study. Disodium hydrogen phosphate dihydrate (Na_2_HPO_4_∙2H_2_O), potassium dihydrogen phosphate (KH_2_PO_4_), NaCl, and trifluoroacetic acid (TFA) were purchased from Merck (Darmstadt, Germany). Formic acid, ammonium bicarbonate, and TPCK-treated trypsin from bovine pancreas were obtained from Sigma-Aldrich (Steinheim, Germany). Furthermore, HPLC SupraGradient acetonitrile (ACN) was obtained from Biosolve (Valkenswaard, The Netherlands) and phosphate-buffered saline (PBS) was made in-house, containing 5.7 g/L Na_2_HPO_4_∙2H_2_O, 0.5 g/L KH_2_PO_4_ and 8.5 g/L NaCl.

## IgG purification and enzymatic digestion

All 206 plasma samples from splenectomized (*n* = 73) and non-splenectomized individuals (*n* = 133) were randomized through four 96-well plates, including blanks (PBS) and two sets of standards: pooled plasma samples, made by pooling random 50 plasma samples from the study and plasma standards purchased from Affinity Biologicals Inc. (VisuCon_F control plasma; Affinity Biologicals Inc., Ancaster, ON, Canada). Five pooled plasma samples were distributed per plate to assess the technical variability of the method. Three plasma standards (VisuCon_F control plasma) were aliquoted per plate as a positive control. Total IgG was affinity-purified by protein G affinity beads (GE Healthcare, Uppsala, Sweden) in 96-well filter plates (0.7 mL wells, PE frit, Orochem, Naperville, IL) as described before.^1^ Briefly, 1 µL of serum were diluted in 20 µL of PBS and incubated with 2 µL of beads for 1 h at room temperature on a plate shaker (1000 rpm; Heidolph Titramax 100; Heidolph, Kelheim, Germany). Five pooled IgG samples and three plasma standards (VisuCon_F control plasma; Affinity Biologicals Inc., Ancaster, ON, Canada) were distributed per plate as a positive control. After washing, IgG was eluted with 100 µL of 100 mM FA and eluates were dried in a centrifugal vacuum concentrator (Martin Christ Gefriertrocknungsanlagen GmbH, Osterode am Harz, Germany) at 50°C for approximately 2.5 h. 40 µL of 25 mM ammonium bicarbonate buffer (pH 8.0) containing 0.25 mg/mL TPCK-treated trypsin were added to the dried total IgG. Samples were dissolved on a shaker at room temperature for 10 min and incubated for 18 h at 37°C.

## Mass spectrometric analysis

The total IgG glycopeptides were analyzed using an Ultimate 3000 RSLC nano liquid chromatography system (Dionex/Thermo Fisher Scientific, Sunnyvale, CA) coupled to a Maxis Impact HD quadrupole time-of-flight (QTOF)-MS instrument (Bruker Daltonics, Bremen, Germany) as described previously ^1^. Briefly, 0.2 µL of IgG glycopeptides were loaded onto an Acclaim PepMap100 C18 trapping column (5 mm x 300 µm i.d., Thermo Fisher Scientific, Sunnyvale, CA) and washed with 100% A (0.1% TFA in water) at a flow rate of 25 µL/min. Subsequently, the tryptic (glyco-)peptides were separated on a nanoEase M/Z Peptide BEH C18 analytical column (100 mm x 75 µm i.d., 1.7 µm, 130 Å, 1/PK, Waters) with a flow rate of 0.6 µL/min. The following linear gradient of solvent A (0.1% TFA in water) and solvent B (95% acetonitrile) was applied: 0 min 3% B, 4.5 min 21.7% B, 5.5 min 50% B, 8 min 50% B, 9 min 3% B, 11.5 min 3% B. Electrospray ionization was achieved in a CaptiveSpray source operating in the positive ion mode and enhanced using the nanoBooster technology (Bruker Daltonics, Bremen, Germany). The solvent was evaporated at 180°C utilizing a nitrogen stream of 3 L/min saturated with acetonitrile. Mass spectra were acquired from *m/z* 550 to 1800 at a frequency of 1 Hz.

## Data processing

Initially, the liquid chromatography datasets were manually verified using Data Analysis version 5.0 software (Bruker Daltonik). Using the described separation method, glycopeptides were separated based on the polypeptide backbone of the three subclass-specific types of tryptic IgG *N-*glycopeptides, such as IgG1, IgG4 and indistinguishable IgG2 and IgG3 (IgG1: EEQYNSTYR, IgG2: EEQFNSTFR, IgG4: EEQFNSTYR). The LC-MS datasets were converted into the mzXML file format using MSConvert (ProteoWizard 3.0 suite). Based on literature, a list of pre-defined glycan features with theoretical *m/z* values was compiled and manually completed by the identification of the remaining glycoform in MS^1^ sum spectra. The list consisted of 19 IgG1 glycopetides, 10 IgG2 glycpeptides and 8 IgG4 glycopeptides (**Table S1**). For glycosylation profiling, the retention times of the clusters of differentially glycosylated variants of each IgG subclass were identified using MZmine version 2.33 software. The in-house developed software LaCyTools version 1.1.0 was used for alignment, generation of sum spectra, calibration and extraction of the data.^2^ Targeted alignment of the time axis of the chromatograms was done using the defined alignment features (**Table S1**). Sum spectra were generated per glycopeptide cluster. Calibration of sum spectra was performed based on a list of manually compiled calibration features and their retention times (**Table S1**). For the extraction for glycopeptide signal intensities, the calibrated sum spectra covering at least 90% of the theoretical isotopic pattern were used for area integration. The integrated signal intensities were extracted in both 2+ and 3+ charge states, within an *m/z* window of ±0.1 Th around each isotopic peak and time window ±16 s around the retention time. Further, quality control (QC) parameters of each signal per charge state, such as the mass accuracy (< 20 ppm deviation), the signal to noise ratio (S/N > 9), and the isotopic pattern quality score (IPQ < 0.2) were used to perform analyte curation and discard low-quality analytes. For each glycopeptide passing the analyte inclusion criteria, the signal intensities of double and triple charge state were summed. For spectral QC, a 2% threshold of the total intensity and minimum number of analytes was determined per four biological groups (healthy individuals, ITP patients, splenectomized healthy individuals, splenectomized ITP patients). The spectra with the lowest number of analytes and with extreme total intensity values exceeding the threshold were excluded from the dataset. Out of 206 total samples, four analysis were excluded from each subclass due to low spectral quality — all control samples, except one trauma splenectomized patient dataset for IgG4. The single *N*-glycopeptide intensities within one IgG subclass were corrected for the isotopic fraction integrated and normalized to the total signal intensity per this glycopeptide cluster, resulting in relative intensity of each analyte per IgG subclass. The derived glycosylation traits were calculated (**Table S2**). Repeatability of the method was assessed by measuring a coefficient of variation (CV) of the pooled plasma standard technical replicates.

# **Supplementary Tables**

Table S1. The list of curated glycosylation compositions detected by nano-LC-ESI-MS and the corresponding m/z values of exact masses of human IgG Fc glycopeptides. (*) Glycan compositions used for alignment, (bolded) Glycan structural feature used for calibration.

| **IgG1 P01857*** | | | **IgG2 P01859*** | | | **Ig4 P01861*** | | |
| --- | --- | --- | --- | --- | --- | --- | --- | --- |
| **glycoform** | **[M+2H]^2+^** | **[M+3H]^3+^** | **glycoform** | **[M+2H]^2+^** | **[M+3H]^3+^** | **glycoform** | **[M+2H]^2+^** | **[M+3H]^3+^** |
| H3N3F1 | 1215.99 | 810.99 | H3N3F1 | 1199.99 | 800.33 | H5N4 | 1398.55 | 932.70 |
| **H3N4** | 1244.50 | 830.00 | **H3N4F1*** | 1301.53 | 868.02 | **H3N4F1*** | 1309.53 | 873.36 |
| H4N3F1 | 1297.01 | 865.01 | H4N4 | 1309.53 | 873.36 | **H4N4F1*** | 1390.56 | 927.37 |
| **H3N4F1*** | 1317.53 | 878.69 | **H4N4F1*** | 1382.56 | 922.04 | H3N5F1 | 1411.07 | 941.05 |
| H4N4 | 1325.52 | 884.02 | H3N5F1 | 1403.07 | 935.72 | **H5N4F1** | 1471.58 | 981.39 |
| H3N5 | 1346.04 | 897.69 | **H5N4F1** | 1463.58 | 976.06 | H4N4F1S1 | 1536.10 | 1024.40 |
| **H4N4F1*** | 1398.55 | 932.70 | **H4N5F1** | 1484.10 | 989.73 | H5N4S1 | 1544.10 | 1029.74 |
| H5N4 | 1406.55 | 938.04 | H4N4F1S1 | 1528.11 | 1019.07 | **H5N4F1S1*** | 1617.13 | 1078.42 |
| H3N5F1 | 1419.07 | 946.38 | H5N5F1 | 1565.12 | 1043.75 |  |  |  |
| H4N5 | 1427.06 | 951.71 | **H5N4F1S1*** | 1609.13 | 1073.09 |  |  |  |
| **H5N4F1** | 1479.58 | 986.72 |  |  |  |  |  |  |
| **H4N5F1** | 1500.09 | 1000.40 |  |  |  |  |  |  |
| H5N5 | 1508.09 | 1005.73 |  |  |  |  |  |  |
| H4N4F1S1 | 1544.10 | 1029.74 |  |  |  |  |  |  |
| H5N4S1 | 1552.10 | 1035.07 |  |  |  |  |  |  |
| H5N5F1 | 1581.12 | 1054.42 |  |  |  |  |  |  |
| **H5N4F1S1*** | 1625.13 | 1083.75 |  |  |  |  |  |  |
| H5N5F1S1 | 1726.67 | 1151.45 |  |  |  |  |  |  |
| H5N4F1S2 | 1770.67 | 1180.79 |  |  |  |  |  |  |

Table S2. Derived trait calculation. The compositions are placeholders for their respective relative intensities.

| **Derived trait** | **Description** | **Calculation** |
| --- | --- | --- |
| IgG1 Bisection | Bisection on IgG1 | (IgG1_H3N5 + IgG1_H3N5F1 + IgG1_H4N5 + IgG1_H4N5F1 + IgG1_H5N5 + IgG1_H5N5F1 + IgG1_H5N5F1S1) / (IgG1_H3N3F1 + IgG1_H3N4 + IgG1_H4N3F1 + IgG1_H3N4F1 + IgG1_H4N4 + IgG1_H3N5 + IgG1_H4N4F1 + IgG1_H5N4 + IgG1_H3N5F1 + IgG1_H4N5 + IgG1_H5N4F1 + IgG1_H4N5F1 + IgG1_H5N5 + IgG1_H4N4F1S1 + IgG1_H5N4S1 + IgG1_H5N5F1 + IgG1_H5N4F1S1 + IgG1_H5N5F1S1 + IgG1_H5N4F1S2) |
| IgG1 Fucosylation | Fucosylation on IgG1 | (IgG1_H3N3F1 + IgG1_H4N3F1 + IgG1_H3N4F1 + IgG1_H4N4F1 + IgG1_H3N5F1 + IgG1_H5N4F1 + IgG1_H4N5F1 + IgG1_H4N4F1S1 + IgG1_H5N5F1 + IgG1_H5N4F1S1 + IgG1_H5N5F1S1 + IgG1_H5N4F1S2) / (IgG1_H3N3F1 + IgG1_H3N4 + IgG1_H4N3F1 + IgG1_H3N4F1 + IgG1_H4N4 + IgG1_H3N5 + IgG1_H4N4F1 + IgG1_H5N4 + IgG1_H3N5F1 + IgG1_H4N5 + IgG1_H5N4F1 + IgG1_H4N5F1 + IgG1_H5N5 + IgG1_H4N4F1S1 + IgG1_H5N4S1 + IgG1_H5N5F1 + IgG1_H5N4F1S1 + IgG1_H5N5F1S1 + IgG1_H5N4F1S2) |
| IgG1 Galactosylation | Galactosylation per antenna of diantennary glycans on IgG1 | (1/2 * (IgG1_H4N3F1 + IgG1_H4N4 + IgG1_H4N4F1 + IgG1_H4N5 + IgG1_H4N5F1 + IgG1_H4N4F1S1) + (IgG1_H5N4 + IgG1_H5N4F1 + IgG1_H5N5 + IgG1_H5N4S1 + IgG1_H5N5F1 + IgG1_H5N4F1S1 + IgG1_H5N5F1S1 + IgG1_H5N4F1S2)) / (IgG1_H3N3F1 + IgG1_H3N4 + IgG1_H4N3F1 + IgG1_H3N4F1 + IgG1_H4N4 + IgG1_H3N5 + IgG1_H4N4F1 + IgG1_H5N4 + IgG1_H3N5F1 + IgG1_H4N5 + IgG1_H5N4F1 + IgG1_H4N5F1 + IgG1_H5N5 + IgG1_H4N4F1S1 + IgG1_H5N4S1 + IgG1_H5N5F1 + IgG1_H5N4F1S1 + IgG1_H5N5F1S1 + IgG1_H5N4F1S2) |
| IgG1 Sialylation | Sialylation per antenna of diantennary glycans on IgG1 | (1/2 * (IgG1_H4N4F1S1 + IgG1_H5N4S1 + IgG1_H5N4F1S1 + IgG1_H5N5F1S1) + (IgG1_H5N4F1S2)) / (IgG1_H3N3F1 + IgG1_H3N4 + IgG1_H4N3F1 + IgG1_H3N4F1 + IgG1_H4N4 + IgG1_H3N5 + IgG1_H4N4F1 + IgG1_H5N4 + IgG1_H3N5F1 + IgG1_H4N5 + IgG1_H5N4F1 + IgG1_H4N5F1 + IgG1_H5N5 + IgG1_H4N4F1S1 + IgG1_H5N4S1 + IgG1_H5N5F1 + IgG1_H5N4F1S1 + IgG1_H5N5F1S1 + IgG1_H5N4F1S2) |
| IgG1 Sialylation per galactose | Sialylation per galactose of diantennary glycans on IgG1 | IgG1 Sialylation / IgG1 Galactosylation |
| IgG2/3 Bisection | Bisection on IgG2/3 | (IgG2/3_H3N5F1 + IgG2/3_H4N5F1 + IgG2/3_H5N5F1) / (IgG2/3_H3N3F1 + IgG2/3_H3N4F1 + IgG2/3_H4N4 + IgG2/3_H4N4F1 + IgG2/3_H3N5F1 + IgG2/3_H5N4F1 + IgG2/3_H4N5F1 + IgG2/3_H4N4F1S1 + IgG2/3_H5N5F1 + IgG2/3_H5N4F1S1) |
| IgG2/3 Fucosylation | Fucosylation on IgG2/3 | (IgG2/3_H3N3F1 + IgG2/3_H3N4F1 + IgG2/3_H4N4F1 + IgG2/3_H3N5F1 + IgG2/3_H5N4F1 + IgG2/3_H4N5F1 + IgG2/3_H4N4F1S1 + IgG2/3_H5N5F1 + IgG2/3_H5N4F1S1) / (IgG2/3_H3N3F1 + IgG2/3_H3N4F1 + IgG2/3_H4N4 + IgG2/3_H4N4F1 + IgG2/3_H3N5F1 + IgG2/3_H5N4F1 + IgG2/3_H4N5F1 + IgG2/3_H4N4F1S1 + IgG2/3_H5N5F1 + IgG2/3_H5N4F1S1) |
| IgG2/3 Galactosylation | Galactosylation per antenna of diantennary glycans on IgG2/3 | (1/2 * (IgG2/3_H4N4 + IgG2/3_H4N4F1 + IgG2/3_H4N5F1 + IgG2/3_H4N4F1S1) + (IgG2/3_H5N4F1 + IgG2/3_H5N5F1 + IgG2/3_H5N4F1S1)) / (IgG2/3_H3N3F1 + IgG2/3_H3N4F1 + IgG2/3_H4N4 + IgG2/3_H4N4F1 + IgG2/3_H3N5F1 + IgG2/3_H5N4F1 + IgG2/3_H4N5F1 + IgG2/3_H4N4F1S1 + IgG2/3_H5N5F1 + IgG2/3_H5N4F1S1) |
| IgG2/3 Sialylation | Sialylation per antenna of diantennary glycans on IgG2/3 | (1/2 * (IgG2/3_H4N4F1S1 + IgG2/3_H5N4F1S1)) / (IgG2/3_H3N3F1 + IgG2/3_H3N4F1 + IgG2/3_H4N4 + IgG2/3_H4N4F1 + IgG2/3_H3N5F1 + IgG2/3_H5N4F1 + IgG2/3_H4N5F1 + IgG2/3_H4N4F1S1 + IgG2/3_H5N5F1 + IgG2/3_H5N4F1S1) |
| IgG2/3 Sialylation per galactose | Sialylation per galactose of diantennary glycans on IgG2/3 | IgG2/3 Sialylation / IgG2/3 Galactosylation |
| IgG4 Bisection | Bisection on IgG4 | (IgG4_H3N5F1) / (IgG4_H5N4 + IgG4_H3N4F1 + IgG4_H4N4F1 + IgG4_H3N5F1 + IgG4_H5N4F1 + IgG4_H4N4F1S1 + IgG4_H5N4S1 + IgG4_H5N4F1S1) |
| IgG4 Galactosylation | Galactosylation per antenna of diantennary glycans on IgG4 | (1/2 * (IgG4_H4N4F1 + IgG4_H4N4F1S1) + (IgG4_H5N4 + IgG4_H5N4F1 + IgG4_H5N4S1 + IgG4_H5N4F1S1)) / (IgG4_H5N4 + IgG4_H3N4F1 + IgG4_H4N4F1 + IgG4_H3N5F1 + IgG4_H5N4F1 + IgG4_H4N4F1S1 + IgG4_H5N4S1 + IgG4_H5N4F1S1) |
| IgG4 Fucosylation | Fucosylation on IgG4 | (IgG4_H3N4F1 + IgG4_H4N4F1 + IgG4_H3N5F1 + IgG4_H5N4F1 + IgG4_H4N4F1S1 + IgG4_H5N4S1 + IgG4_H5N4F1S1) / (IgG4_H5N4 + IgG4_H3N4F1 + IgG4_H4N4F1 + IgG4_H3N5F1 + IgG4_H5N4F1 + IgG4_H4N4F1S1 + IgG4_H5N4S1 + IgG4_H5N4F1S1) |
| IgG4 Sialylation | Sialylation per antenna of diantennary glycans on IgG4 | (1/2 * (IgG4_H4N4F1S1 + IgG4_H5N4S1 + IgG4_H5N4F1S1)) / (IgG4_H5N4 + IgG4_H3N4F1 + IgG4_H4N4F1 + IgG4_H3N5F1 + IgG4_H5N4F1 + IgG4_H4N4F1S1 + IgG4_H5N4S1 + IgG4_H5N4F1S1) |
| IgG4 Sialylation per galactose | Sialylation per galactose of diantennary glycans on IgG4 | IgG4 Sialylation / IgG4 Galactosylation |

# **Supplementary Figures**

Figure S1.


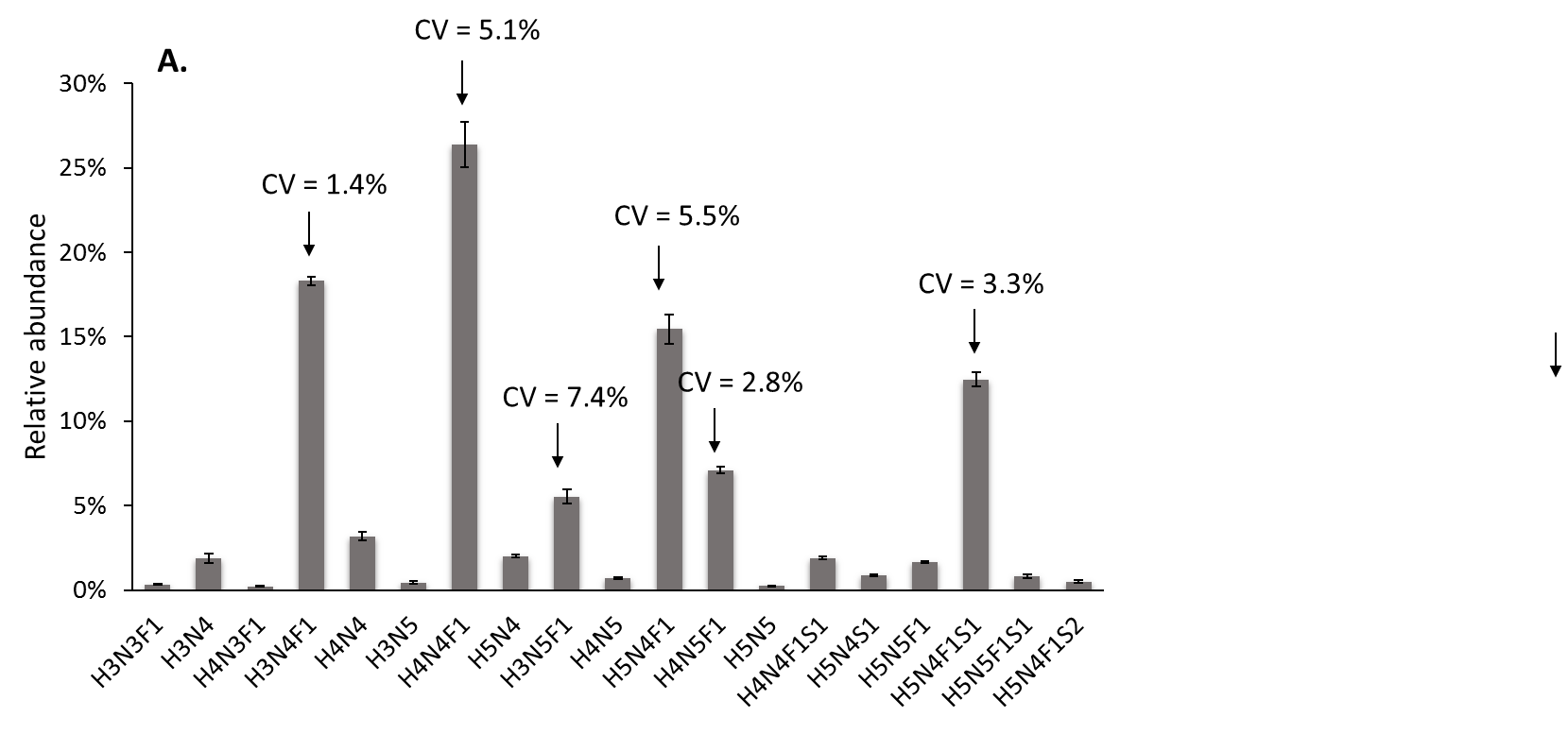

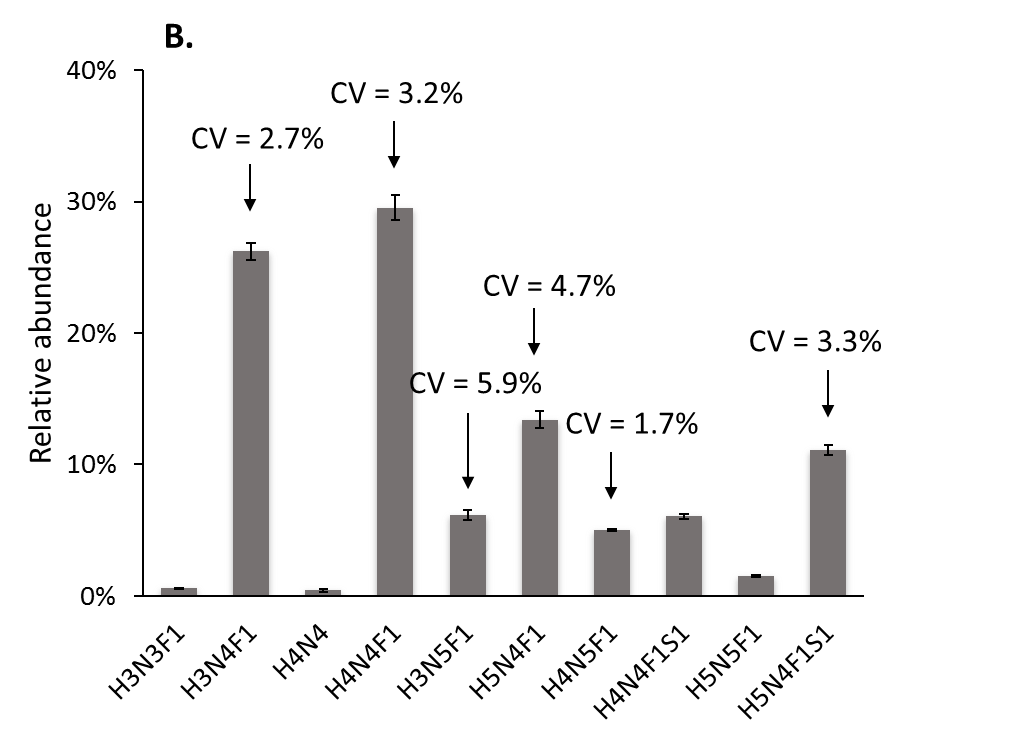


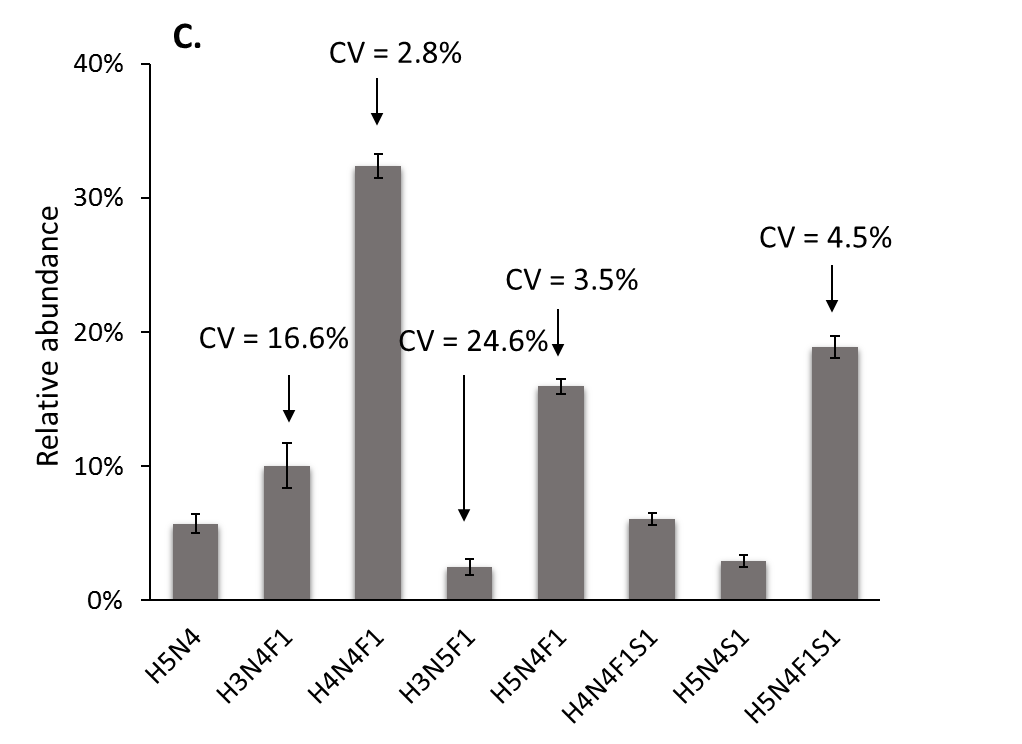


Figure S1. Repeatability of the method. Average relative abundances of A. IgG1, B. IgG2/3 and C. IgG4 glycopeptides from 24 replicates of a pooled plasma sample. Coefficient of variations (CV) is displayed for six prominent glycoforms on IgG (H3N4F1, H4N4F1, H3N5F1, H5N4F1, H4N5F1, H5N4F1S1). For those glycoforms a median coefficient of variation of 4.3%, 3.6% and 10.4% was observed for IgG1, IgG2/3 and IgG4, respectively. The error bars represent the standard deviation of the mean.

Figure S2.

**
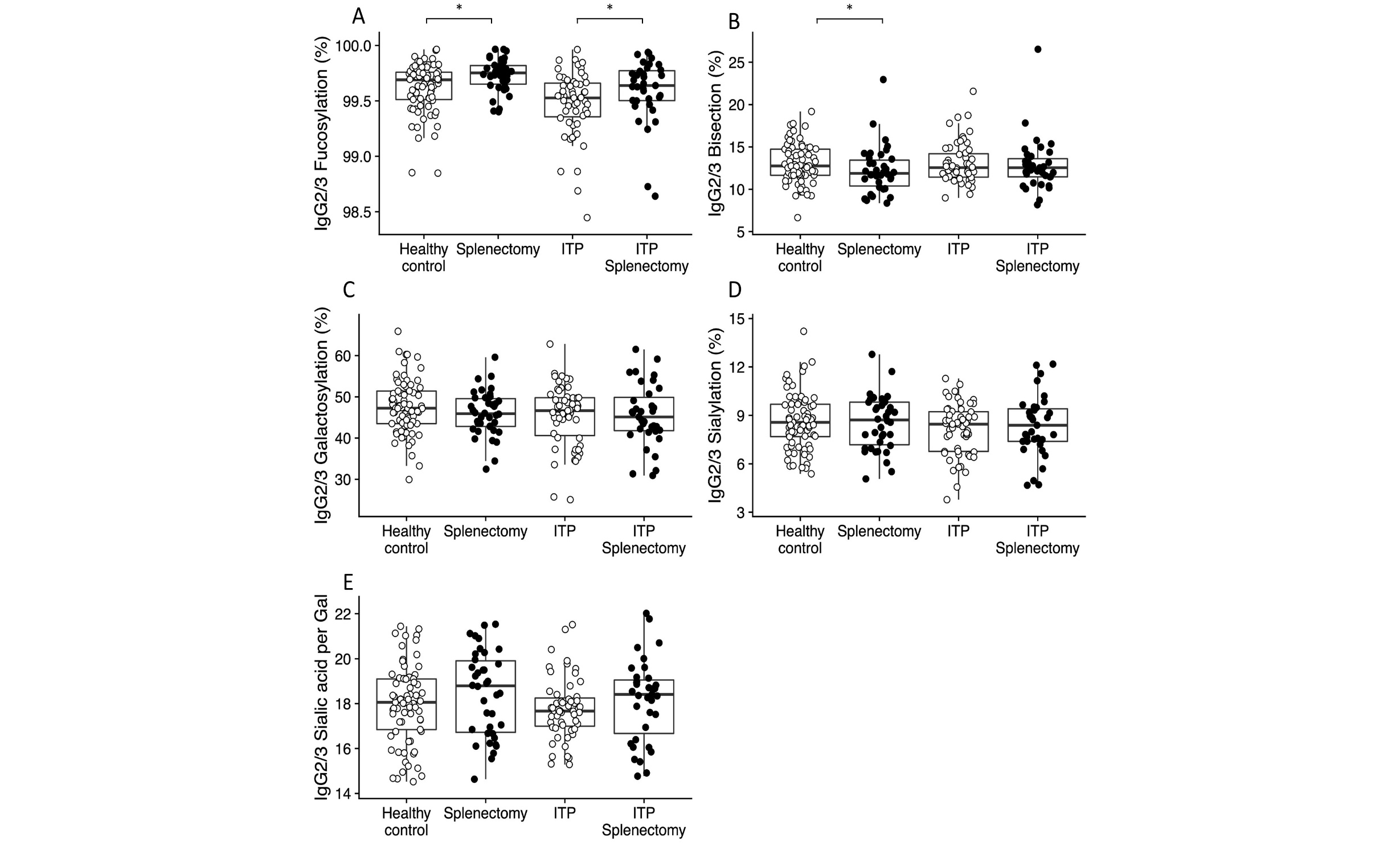
**

**Figure** S2. Comparison of IgG2/3-Fc glycosylation profiles **in regard to fucosylation, bisection, galactosylation, sialylation and sialic acid per galactose after splenectomy in healthy individuals and ITP patients.** Compared to the respective controls, IgG2/3 Fc fucosylation was higher in splenectomized and otherwise healthy individuals (P = 0.006; Wilcoxon-rank sum test), as well as in splenectomized ITP patients (P = 0.016; Wilcoxon-rank sum test). Regarding IgG2/3 Fc bisection, otherwise healthy individuals showed lower levels (P = 0.042; Wilcoxon-rank sum test), whereas in splenectomized ITP patients this was difference was less prominent and not significant. All other glycosylation features were not significantly different between the groups.

Figure S3.


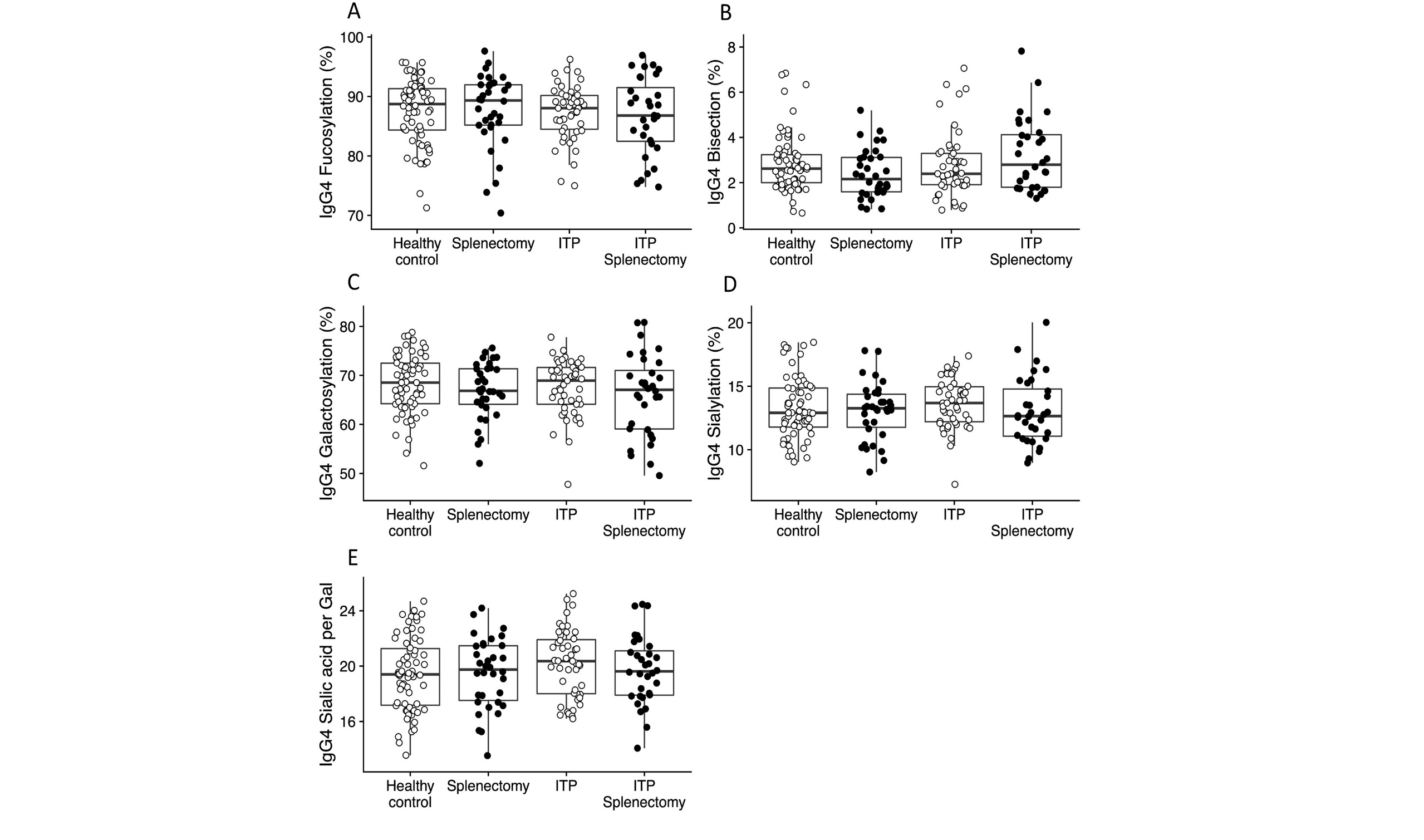


Figure S3. Comparison of IgG4-Fc glycosylation profiles **in regard to fucosylation, bisection, galactosylation, sialylation and sialic acid per galactose after splenectomy in healthy individuals and ITP patients.** All glycosylation features were not significantly different between the groups.

# References

1 Falck, D., Jansen, B. C., de Haan, N. & Wuhrer, M. High-Throughput Analysis of IgG Fc Glycopeptides by LC-MS. *Methods Mol Biol* **1503**, 31-47, (2017).

2 Jansen, B. C. *et al.* LaCyTools: A Targeted Liquid Chromatography-Mass Spectrometry Data Processing Package for Relative Quantitation of Glycopeptides. *J Proteome Res* **15**, 2198-2210, (2016).
